# High-fidelity simulation for mental health nurse residents: a mixed-methods study of communication competencies and learning perceptions

**DOI:** 10.3389/fmed.2025.1612777

**Published:** 2025-11-03

**Authors:** Julio González Luis, Jesús Sánchez Adsuara, Almudena Medrano Andrés, Inmaculada Corral Liria, Marta Losa Iglesias, Miriam Alonso Maza

**Affiliations:** ^1^Gregorio Marañón Hospital, Madrid, Spain; ^2^Ramón y Cajal University Hospital, Madrid, Spain; ^3^Department of Nursing and Stomatology, Rey Juan Carlos University, Móstoles, Spain

**Keywords:** clinical simulation, mental health, nurses, communication, suicide

## Abstract

**Background:**

High-fidelity clinical simulation is increasingly used in postgraduate nursing education, particularly in mental health training, where it can support the acquisition of competencies for managing complex situations such as suicidal behavior.

**Objective:**

To explore the perceptions and learning outcomes of mental health nursing residents during high-fidelity simulation sessions, with a focus on communication, affective context, and professional competencies.

**Methods:**

A mixed study was conducted with a qualitative core design and a complementary quantitative component. Sixty-nine first- and second-year mental health nursing residents from the Community of Madrid participated in simulation sessions structured according to the MAES© methodology. Qualitative data (focus groups, written reports, field notes) were analyzed thematically by three independent researchers, ensuring rigor through triangulation, member checking, and reflexivity. Quantitative contextual data were obtained through a checklist of nursing interventions and descriptive statistics.

**Results:**

The analysis generated three overarching themes: *Communication style* (residents emphasized active listening and the importance of non-verbal language; e.g., “Sometimes silence says more than words”), *Affective context* (awareness of their own emotional impact during patient interactions), and *Training, knowledge, and experience* (residents identified gaps and valued simulation as a safe environment to bridge them). Quantitative results supported these findings but were interpreted only as contextual information.

**Conclusions:**

High-fidelity simulation promotes the integration of theoretical knowledge into practice, strengthens self-confidence, and contributes to safer management of suicidal behavior in clinical contexts. The findings support the transferability of simulation-based training to mental health practice and highlight the need for longitudinal research to evaluate its impact on real patient outcomes.

## Introduction

According to the World Health Organization (WHO), suicide represents a serious public health problem worldwide (1). This concern includes not only completed suicides but also suicidal ideation and previous suicide attempts. The high mortality rate of suicide suggests the need for a broad research approach. This approach should involve professionals from various social health fields, enabling an in-depth understanding of the phenomenon and the exploration of related experiences (2, 3).

Clinical simulations are based on the recreation of real systems or situations and provide guided and immersive experiences that replicate aspects of the clinical environment in an interactive manner (4). In the field of mental health, high-fidelity simulation gives nurses the opportunity to interact with patients suffering from various psychiatric pathologies, helping the nurses overcome their own fears and interpersonal barriers and facilitating a more natural and standardized approach to these patients. This educational methodology allows students to evaluate their clinical reasoning skills and improve decision-making. Several studies indicate that simulations with standardized patients can reduce students' anxiety and increase their confidence before facing clinical practice in mental health. These simulations not only improve clinical skills and therapeutic communication but also aid in the management of different mental disorders, the development of empathy and the evaluation of competencies. Similarly, simulations are suggested to have a potential benefit in the development of nursing students' attitudes toward mental health problems (5–7).

Training residents in mental health nursing became an official speciality with the regulation of specialties in 1998, which progressively increased the number of places offered. The teachers and collaborators have the mission of training residents in diagnostic, technical, communication and ethical skills through care activities, clinical sessions, workshops and tutored supervision. Although clinical simulation is recognized for its ability to foster self-confidence and critical thinking, its regular use in resident training remains limited (8, 9).

## Theoretical framework

Highly realistic clinical simulations provide opportunities for experiential and reflective learning, and their use has spread globally in the training of health care professionals. The strategies used to carry out simulations effectively, including the preparation of simulated scenarios obtained from specialized bibliographies, to address specific skills of students are well documented (10). However, there is little evidence on the use of simulations in the training of mental health resident nurses to address suicidal behaviors. Given the increasing requirement in health services to address current and highly relevant issues such as suicidal ideation, it is necessary to evaluate the effects of simulation training, including the acquisition of competency, in these professionals. The objectives of this study were to explore the perceptions of mental health resident nurses regarding high-fidelity simulation sessions related to the management of patients with suicidal behavior and to improve the acquisition of the necessary nursing competencies for the management of these patients.

While high-fidelity clinical simulation is well established as an effective educational tool in health professional training, its incorporation into mental health nursing residency programs remains limited, particularly in the management of suicidal behavior. This gap underscores a critical need for research, as most existing studies focus on general nursing or other clinical specialties and overlook the specific training requirements of mental health residents. The present study addresses this gap by examining residents' perceptions of high-fidelity simulation and evaluating its potential to enhance the development of clinical, communicative, and ethical competencies essential for the care of patients at risk of suicide. In doing so, it contributes to the current body of evidence and informs the design of future educational strategies.

## Methods

### Study design

An exhaustive qualitative study was conducted within a hermeneutic phenomenological framework inspired by Heidegger, aiming to explore the depth of human experience from both scientific and humanistic perspectives. This approach goes beyond the mere description of phenomena, seeking instead to uncover the meanings that individuals attribute to their lived reality. In operational terms, the study employed in-depth, semi-structured interviews complemented by systematic coding, categorization, and interpretive analysis. Given the inclusion of quantitative data derived from structured checklists (e.g., execution rates expressed as percentages), the design was defined as mixed, with a predominantly qualitative orientation and a secondary quantitative component, which served to provide contextual support rather than to drive the main analysis. This strategy ensured consistency between the study objectives, the data collection techniques, and the analytic procedures, which combined hermeneutic interpretation with thematic analysis following Braun and Clarke's six-phase model. To guarantee methodological rigor and transparency, the research adhered to international standards for qualitative inquiry, specifically the Consolidated Criteria for Reporting Qualitative Research (COREQ) and the Standards for Reporting Qualitative Research (SRQR) (11–13).

### Sample and environment

Sixty-nine first- and second-year mental health resident nurses (training period stipulated in the Specialized Health Training) from the Community of Madrid, whose training covered the periods of 2021–2022 and 2022–2023, that is, the last two generations of residents, and who participated in high-fidelity simulation sessions were included in this study. The study was conducted between January 1 and February 28, 2024. Intentional nonprobability sampling was applied. The inclusion criteria were as follows: (1) active residents in the first and second years of mental health nursing residency carrying out specialized health training in the Community of Madrid and (2) individuals who signed informed consent forms. The exclusion criteria were resident nurses who (1) left the residency during the data collection period and (2) had a temporary disability (TD) of more than 6 months. The study was carried out within the training period as part of the mandatory weekly lessons; therefore, all the simulation sessions were based on the theory taught during their training, either at a theoretical or practical level.

Participation was framed within the mandatory weekly training schedule, and therefore all simulation sessions were based on theoretical and practical content previously covered in their residency program. The simulations took place in university-affiliated clinical skills laboratories specifically equipped for high-fidelity simulation, with standardized schedules and controlled environmental conditions to minimize interruptions and external interferences. Regarding the researcher-participant relationship, the sessions were moderated by faculty members with formal training in clinical simulation and qualitative research, who adopted the role of facilitators/observers while maintaining a reflexive stance on their own potential biases and epistemic position. Field notes were systematically taken to complement the analysis. Theoretical saturation was assessed iteratively during the analysis of the simulation debriefings and focus groups (9–10 participants per group), and was considered achieved when no new categories or themes emerged.

To strengthen rigor and traceability, data were transcribed verbatim and managed with qualitative analysis software (NVivo), which allowed for an organized coding process and the maintenance of an audit trail.

### High-fidelity simulation sessions

All the clinical simulation sessions were designed according to the six stages proposed by the MAES © methodology (14) as follows: (1) selection of work teams and the establishment of a group identity through structured and guided group dynamics; (2) voluntary choice of the subject of study from cases taken from reality or fiction; (3) establishment of baseline competencies and development of the competency curriculum via a joint brainstorming session; (4) design of a clinical simulation scenario by the students in which the competency goals are exploited; (5) execution of the simulated clinical experience; and (6) debriefing and presentation of the acquired competencies. In this study, (1) the resident nurses in mental health were divided into work teams composed of 2–3 residents (a total of 34–35 work groups) building their own group identity; (2) the issue to address in the simulation was identified; (3) a baseline was developed in terms of nursing competencies; (4) the simulated scenarios were developed; (5) the scenarios were carried out with professional actors with experience in simulation; and (6) the simulation concluded with a debriefing session.

The main researcher and collaborators provided a brief description of the simulated scenario to each work team before the simulation experience, allowing them to identify the Nursing Interventions Classification (NIC) and determine five nursing activities required to resolve each simulated scenario. The research team designed a checklist to evaluate the acquisition of competencies of the mental health nurses on each work team (Table 1). A total of 34–35 simulation sessions were designed, one for each work team (one or two participants for each simulation scenario). The learning objectives covered by each simulation session were as follows: (1) appropriate selection of the NICs and their related nursing activities for the resolution of the simulated scenario and (2) adequate performance of the necessary nursing competencies for the proper management of suicidal behavior during the simulation scenario. The NIC-based checklist was subjected to a process of content validation through consultation with a panel of experts in mental health nursing and clinical simulation using a modified Delphi technique, followed by pilot testing with a subset of residents, thus ensuring its reliability and applicability in the evaluation of nursing competencies during high-fidelity simulation.

**Table 1:** Checklists of the most important NICs and their nursing activities selected by the majority of the resident nurses in each simulated scenario.

**Simulated scenario**	**Suicidal behavior**	**NIC and nursing activities**	**Checklist (yes/no)**
SCENARIO A 30-year-old patient with active suicidal ideation including a structured plan who presents to emergency services with marked anguish.	Life situation	**[5250] Support in decision-making**	
		(a) Help the patient clarify the values and expectations used to make fundamental life decisions.	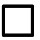
		(b) Inform the patient about alternative points of view and solutions clearly and with full support.	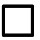
		(c) Serve as a link between the patient and the family.	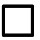
		(d) Establish communication with the patient at the time of admission.	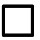
		(e) Serve as a link between the patient and other health professionals.	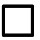
SCENARIO B 20-year-old patient who comes to the Mental Health Center for unstructured ideas of death due to a job dismissal and a breakup.	Impulsiveness and aggression	**[5330] Control of mood**	
		(a) Determine if the patient poses a risk to the safety of himself and others.	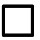
		(b) Implement the necessary precautions to safeguard the patient and those around him from the risk of physical harm (suicide, self-injury, running away, violence).	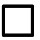
		(c) Consider the possibility of hospitalization if the patient exhibits an altered mood that poses safety risks, is unable to meet the needs of self-care and/or lacks social support.	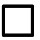
		(d) Provide or refer to psychotherapy (cognitive-behavioral, interpersonal, couple, family, group therapy), when appropriate.	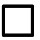
		e) Teach new coping and problem-solving techniques.	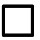

During each simulated scenario, which lasted 7–10 min, the clinical performance of each work team was monitored by three direct observers. Actors played the role of the patient in each simulated scenario (acting as standardized patients according to the clinical simulation methodology). All of these actors were trained to ensure high-fidelity experience and a standardized process. After each scenario was completed, all the residents discussed and analyzed their clinical performance during a 60-min debriefing session. The principal investigator conducted this session via the debriefing tool proposed by Phrampus and O'Donnell (15), the GAS method: Gather, Analyse, and Summarize. Notably, all the requirements proposed by the INACSL Best Practice Standards: SimulationSM (16) were followed to carry out all the high-fidelity simulation sessions.

### Data collection

**Figure 1:**
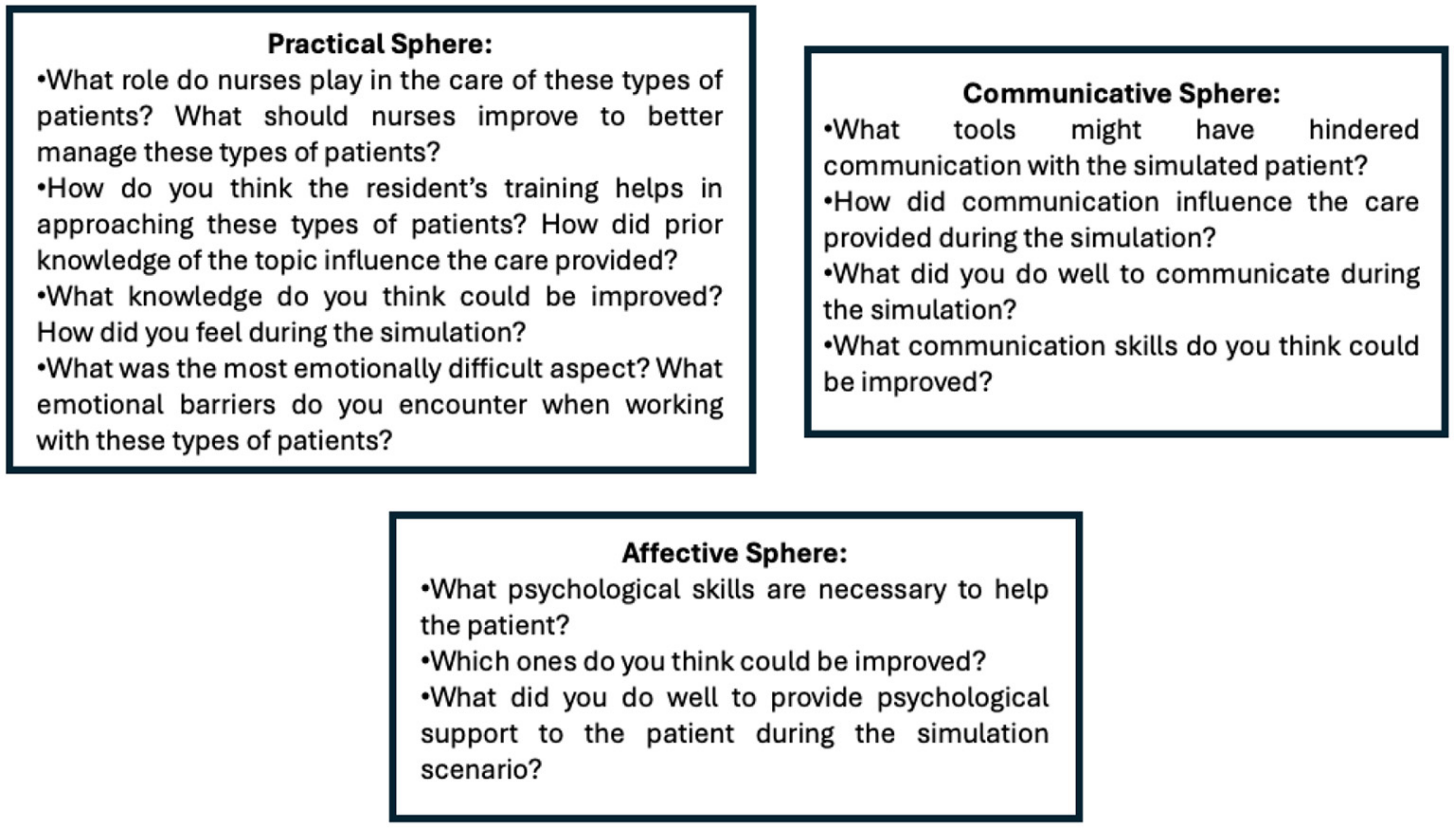
Semistructured script of questions in the stories and discussion groups.

The research team reviewed the NICs and the nursing activities selected by each work team before carrying out the corresponding simulated scenario. The acquisition of nursing competencies, including the five most important nursing activities included in the corresponding NIC and selected by each work team, was evaluated via a checklist (“Yes/No”) (Table 1). During each simulated scenario, three direct observers verified whether the participating resident nurses performed the selected nursing activities. After the clinical simulation sessions, 6–7 focus groups (FGs) were formed, consisting of 9–10 residents, with an average session duration of 60 min. Residents were asked to write stories, and field notes were collected. The FGs were led by a moderator and an observer. The moderator posed questions that had been requested in the reports and were discussed among the mental health resident nurses to determine the acquisition of clinical skills, whereas the observer took field notes on non-verbal aspects of the session and collaborated in the collection and custody of student reports. During the debriefing phase, three relevant themes were identified: communication skills, psychological resources and integration of knowledge and aptitude applied by mental health residents during the simulation sessions. These themes were explored via semistructured questions to guide the stories and later discussed in the FGs (Figure 1).

### Data analysis

Descriptive statistics of the quantitative sociodemographic data and the acquisition of nursing competencies were calculated using IBM SPSS Statistics software, version 28.0 for Windows. For the qualitative component, an exploratory study was carried out within an interpretive theoretical framework, applying thematic analysis as described by Braun and Clarke (17). The analysis was conducted independently by three researchers with prior experience in qualitative research. The procedure included multiple stages: (1) identification of significant words, phrases, and metaphors in the transcripts; (2) reduction of data into meaningful units; (3) organization of these units into clusters of common meaning; and (4) development of subthemes and themes. To ensure transparency, a codebook was generated that illustrated the progression from initial codes → subthemes → themes, accompanied by representative quotations. These quotations were explicitly linked to their corresponding subtheme/theme, thus increasing analytical density and variation.

Triangulation was performed at different levels: of data (written reports, focus groups, and field notes), of researchers (independent coding by three analysts, followed by consensus meetings), and of methods (integration of simulation debriefings and qualitative interviews). Credibility was further enhanced through participant validation (*member checking*), whereby residents were invited to review and confirm the accuracy of the interpretations. Reflexivity was ensured by maintaining reflective journals and positionality statements from the research team, which allowed the identification and control of potential biases.

Study rigor was strengthened through adherence to COREQ and SRQR guidelines (18, 19), providing detailed information on the researchers, participants, sampling strategies, data collection procedures, and analytic process. Replicability was supported by the systematic documentation of each stage and the maintenance of an audit trail using NVivo software.

### Ethical considerations

The study received the approval of the corresponding Research Ethics Committee (approval code: SCSM_ENF). The simulation sessions were carried out as a complementary activity to students' postgraduate training (voluntary activity); however, the mental health resident nurses voluntarily completed the scales before and after the simulation. Although the simulation sessions were embedded in the postgraduate training program, participation in the research component (completion of pre- and post-simulation scales, inclusion of data in the study) was strictly voluntary. Residents could withdraw at any time without any impact on their academic training.

To participate, each resident signed an informed consent form, which detailed the objectives of the research and their acceptance to participate in the study. The study followed the principles and ethical guidelines for medical research with human subjects adopted from the Declaration of Helsinki. The provisions of Organic Law 3/2018 of December 5 on the protection of personal data and guarantee of digital rights were followed for the treatment, communication and transfer of personal data of the participants in the study. Accordingly, the study subjects could contact the main researcher to access, modify or withdraw their data, thus exercising their right to do so. Data treatment proceeded with absolute confidentiality, making it impossible to associate participants with the results of the study, according to Law 14/2007 of July 3 on biomedical research (20, 21).

## Results

### Quantitative results

Sixty-nine resident nurses in mental health participated in the high-fidelity simulation sessions. All the residents voluntarily consented to participate in the study (100% response rate). Most of the participants were women (*n* = 57; 82.6%). The age of the participants ranged from 23 to 46 years (mean = 26.52; SD = 4.31). The research team confirmed that the nursing residents had made an appropriate selection to adequately manage the patient's approach to suicidal behavior, according to each simulation scenario (Table 2). Quantitative results are included because they strengthen the sample used in the qualitative research and provide a comprehensive overview of the study.

**Table 2:** Achievement percentages of the most important NICs and their corresponding nursing activities selected by the majority of the resident nurses in each simulated scenario.

**Scenario A**			**Scenario B**		
**[5250] Support in decision-making**	**% Yes**	**% No**	**[5330] Control of mood**	**% Yes**	**% No**
(a) Help the patient clarify the values and expectations used to make fundamental life decisions.	87%	13%	(a) Determine if the patient poses a risk to the safety of himself and others.	72	28
(b) Inform the patient about alternative points of view and solutions clearly and with full support.	30%	39%	(b) Implement the necessary precautions to safeguard the patient and those around him from the risk of physical harm (suicide, self-injury, running away, violence).	65	35
(c) Serve as a link between the patient and the family.	20%	48%	(c) Consider the possibility of hospitalization if the patient exhibits an altered mood that poses safety risks, is unable to meet the needs of self-care and/or lacks social support.	26	74
(d) Establish communication with the patient at the time of admission.	47%	21%	(d) Provide or refer to psychotherapy (cognitive-behavioral, interpersonal, couple, family, group therapy), when appropriate.	64	36
(e) Serve as a link between the patient and other health professionals.	23%	46%	(e) Teach new coping and problem-solving techniques.	69	31

Overall, 60% of the resident nurses correctly carried out the nursing activities selected for high-fidelity simulation scenario A; 40% of nurses could not remember how to carry out the selected nursing activities and could not complete them. For scenario B, 85% of the nursing residents correctly carried out the selected nursing activities, and 15% could not remember how to carry out the selected nursing activities and could not complete them.

### Qualitative results

After the qualitative data collected in the reports and FGs were analyzed, three main themes and their corresponding subtopics were identified. Table 3 shows all the main themes and subtopics identified, as well as examples of the main statements from the stories and FGs. The communication skills that were put into practice during the simulation sessions were divided into the following subtopics: (4.1) Communication style, (4.2) Understanding the affective context of the patient, and (4.3) Training, knowledge and experience.

**Table 3:** Topics and subtopics identified after the analysis of the stories and discussion groups.

**Topic**	**Subtopics**	**Stories and FGs**
**4.1. Communication style**	4.1.1. Exploring communication patterns.	“*The communication styles could be improved through adaptation according to the specific context*.” (**R: 2**) “*I think I am consolidating my speaking style when interviewing a patient*.” (**R: 40**) “*I think I need to find my communication style and make it mine*.” (**R: 33**) “*They have allowed the patient to make clarifications and used active listening and therapeutic silences*.” (**FG: 2**) “*The position of the therapists, active listening and validation were the central axis of the interview*.” (**FG: 7**)
	4.1.2. The art of verbal interaction: metacommunication.	“*The tone was adequate during the intervention and adapted to that of the patient.”* (**R: 37**) “*The use of a calm tone of voice to counteract the patient's disturbance […]*” (**R: 20**) “*The fact of working better with silence and non-verbal language*.” (**R: 13**) “It *helped to listen actively to the patient without interrupting her speech in an invasive way*.” (**FG: 6**) “*It seems essential to be able to create a barrier between personal interaction and patient care, since this interaction can be an obstacle in regard to helping them*.” (**FG: 1**)
	4.1.3. Improvement as advancement in the profession.	“*More training in communication styles seems insufficient to me.”* (**R: 17**) “*I should improve my non-verbal language to help me remain calm*.” (**R: 9**) “*Perhaps learning to better direct the clinical interview or explain the role of the specialist nurse*.” (**R: 22**) “*The need to continue working on simulations as an essential element of resident training seems fundamental*.” (**FG: 3**) “*Prioritizing the importance of the interview and conducting it without delay is key in the care of mental health patients*.” (**FG: 2**)
**4.2. Understanding the affective context of the patient**	4.2.1. In-depth look at the emotional landscape of the patient.	“*Empathy, emotional intelligence and self-regulation prevent a traumatic situation from affecting me too strongly and keeping me from giving an adequate response to the patient*.” (**R: 52**)” *Empathy, closeness and support are necessary to be able to read the patient*.” (**R: 45**) “*Analytical skills and communication skills are necessary to address certain aspects of our profession. I think this has helped us understand different patients and recognize our strengths and weaknesses*.” (**FGs: 5**)
	4.2.2. Strategies for a genuine connection.	“*An adequate awareness is necessary for each scenario used; without this, it is not possible to build a solid therapeutic alliance*.” (**R: 56**) “*It is difficult for me to foster a therapeutic bond in such a short time. I understand that it is not the main objective, but assertiveness and skills seem insufficient to connect with these types of patients*.” (**R: 29**) “*In general, it was possible to connect with the patient quickly, showing interest in her problem and transmitting availability to her at all times*.” (**FG: 4**)
	4.2.3. Mapping the emotional terrain.	“*I felt weak, and I did not know what to say without harming the patient, since I do not have a good base, which makes me angry*/” (**R: 49**) “*I think one of the most important things that was done was to acknowledge the emotion of the patient, validate those emotions and offer help.”* (**R: 18**) “*In general, I think we have all done a great job in the simulation. We have used the tools we learned, and our different styles of communication are appreciated. We have used empathy and assertiveness without value judgements and resorted to therapeutic silences when necessary*.” (**FG: 1**)
**4.3**. **Training, knowledge and experience**	4.3.1. Learning journey	“It *helps to work with people with these characteristics every day to approach them from the point of view of the different mental health problems*.” (**R: 30**) “*We must understand the patient as a whole, in clinical practice. Specialist nurses can and should not only make an assessment but also coordinate and manage the case*.” (**R: 11**) “*Work experience helps a lot in daily clinical practice*.” (**R: 24**) “*Theoretical training is essential because it gives you a roadmap or guide to follow when facing a complicated issue or a difficult approach such as suicidal behavior*.” (**FG: 3**)
	4.3.2. From theory to practice: integrating training and experience in health care.	“*The residency provides you with comprehensive training for dealing with mental health patients. Until I started the residency, I think I had no idea how many skills were needed to conduct an interview*.” (**R: 7**) “*I consider that training alongside professionals trained in this case is important to give you tools and skills that would not be possible otherwise*.” (**R: 27**) “*It is essential to continue doing this type of training during residency, in addition to guided tutoring*. “ (**FG: 2**)

R represents the story to which the quote refers and the participant number. FG represents the discussion group and the corresponding number.

### First topic: communication style

Considering the communication skills that have been put into practice during the simulation sessions, three subtopics were observed: (a) exploring communication patterns, (b) the art of verbal interaction: metacommunication and (c) improvement as advancement in the profession.

**(A) Exploring communication patterns**. The stories highlight the various ranges of communication skills from clarification and active listening to therapeutic silences and emotional validation. During the interviews, the nurses in a mental health residency dealt with the clinical scenario appropriately and without prejudice. They identified themselves (name and category) and were able to respond to the initial requirements of the patient. The residents offered a safe space to clarify the patient's care needs.

**(B) The art of verbal interaction: metacommunication**. During the simulated cases, the resident nurses in mental health reported the importance of tone (adapting to the cadence and volume of the patient), body posture (greater proximity between patient and provider), and the use of silence and all the non-verbal context that influences a clinical interview. The nurses described how in some cases, these communication skills limited the professional's own interpersonal anguish, as well as the difficulty of conducting an adequate interview and how, even when starting from general questions and progressing to specific questions, patients were not able to answer or moved to another topic without addressing the question.

**(C) Improvement as advancement in the profession**. During the simulated cases, the residents mentioned the importance of previous training for the generation of different styles of communication. The nurses discussed the lack of training in this field and stated that the improvement of the profession involves reinforcing interview skills.

### Second topic: understanding the affective context of the patient

Considering the affective sphere and the emotions that were practiced during the simulation sessions, three subtopics were observed: (a) an in-depth look: revealing the emotional landscape of the patient, (b) strategies for a genuine connection, and (c) mapping the emotional terrain.

**(A) An in-depth look: revealing the emotional landscape of the patient**. In the stories, all the particularities that make a therapist essential and that enable the establishment of a connection with the patient are highlighted. Thus, during the simulated scenarios, self-regulation and emotional intelligence are discussed as basic tools for reading the needs of the patient. The residents not only understood the context of the simulated patient but also asked appropriate questions to explore the severity of suicidal behavior. The nurses provided support and raised safety concerns.

**(B) Strategies for a genuine connection**. Mentalization, defined as the ability of the nurses to interpret patients' behavior and their own behavior according to various mental states, was discussed during the simulations and FGs. In this study, the residents mentioned initial insecurity when addressing issues such as suicidal behavior (mainly first-year residents). These residents commented on the need for connection, which is difficult to achieve with so little time in the simulated scenario. However, within the stipulated time frame, nurses not only managed to connect with the simulated patient but also provided comfort and transmitted clinical safety.

**(C) Mapping the emotional terrain**. This last subtopic included the insecurities of the therapist and how they influence a clinical interview. Although the residents handled the interviews well and asked questions ranging from deductive to inductive, they reported that they felt weak during the simulation. They discussed the fear of making a mistake during the interview and the possibility that their actions, especially their emotions, could harm the simulated patient.

### Third topic: training, knowledge and experience

Considering the spheres of knowledge vs. training and experience regarding skills that were put into practice during the simulation sessions, two subtopics were observed: (a) the learning journey, i.e., attaining clinical knowledge, and (b) turning theory into practice, i.e., the integration of training and experience in health care.

**(A) The learning journey**. During the FGs, the importance of learning during resident training as a cornerstone for good professionals was discussed. The nurses highlighted the need for this topic to be strengthened in all teaching units. Some residents stated that the training for nurses was almost non-existent, prioritizing psychologists and psychiatrists and ignoring mental health nurses.

**(B) Turning theory into practice: integrating training and experience in health care**. During the FGs, the importance of the mental health specialty was discussed as a basis for the provision of specialized care in the field. Many residents reported that prior to residency, training in communication skills and interviews was minimal.

## Discussion

Simulations in mental health can be a useful educational tool to participate in scenarios in safe simulated learning environments. According to the study carried out by Luebbert et al. (22), although both mannequins and standardized patient modalities are useful for increasing the knowledge of mental health nurses, the standardized simulation of patients has a greater impact on several aspects, including clinical reasoning and communication. This finding reinforces the need to improve simulation training in the mental health field as a teaching tool in postgraduate training. In the same vein, Lockertsen et al. (23) noted that high-fidelity simulations during mental health clinical practice could improve the educational environment and the learning outcomes of nursing students. In their study, the simulation increased the self-awareness and deep reflection of the students and broadened their competence in nursing. However, these investigations focused on students in undergraduate training, with studies of graduate students being insufficient or non-existent.

Regarding studies on more specific aspects of mental health patients, Sahin-Bayindir and Buzlu (24) noted that clinical simulation was statistically effective for the development of student skills in addressing physical health problems of psychiatric patients. In addition, in the qualitative findings, the clinical simulation increased the level of knowledge of the students and improved their physical health assessment skills. Similarly, with a focus on aggression, violence and the clinical deterioration of patients admitted with mental health problems, the study by Young et al. (25) on the effectiveness of high-fidelity immersive simulation education to help interprofessional hospital clinical staff recognize and respond to aggression, violence and clinical deterioration of patients showed that immersive simulations were effective in improving the confidence of the medical staff and nurses of the hospital when responding to incidents of aggression/violence and clinical deterioration of a mental health patient. With respect to the education of nursing students via simulation of specific mental health pathologies of patients, the study by Farooq et al. (26) is notable; in this study, the simulation experience was described as the interaction of students with simulated patients (SPs), who had been asked to manifest symptoms of depression, mania, and schizophrenia. The students' evaluation of the simulation experience was positive, and they suggested integrating it into the mental health nursing course. This result is consistent with the findings of this study and the feedback provided by the resident nurses in mental health, who considered this approach a basic need during their postgraduate training; in addition, this result is supported by studies such as that by Weidlich et al. (27), who reported that standardized patients offered a more realistic experience in the evaluation of various domains in the examination of the patient's mental state and the practice of essential therapeutic communication techniques in psychiatric nursing.

With respect to suicidal behavior, the study conducted by Pedrollo et al. (28) revealed notable approaches to post-intervention and initial support provided to people in grief due to suicide; the study also describes a validated scenario that can be used free of charge for the development of clinical simulations in the training of different professional categories. However, there are very few studies that address training via clinical simulation for patients with active suicidal ideation, whether in the foreground or background, and even fewer in postgraduate training.

### Limitations

It is necessary to continue research in this field and to expand it to other nursing specialties, assessing the communication tools and skills acquired during residency training. However, this study also presents certain limitations that should be acknowledged. One methodological concern is the use of a non-validated checklist, which may affect the reliability of some of the findings. In addition, the exploratory nature of the study and the limited sample size restrict the generalizability of the results. While the potential positive impact of high-fidelity simulation in mental health nursing education is noteworthy, this aspect has been emphasized in the discussion. In the limitations section, it is important to adopt a more self-critical stance and recognize the intrinsic weaknesses of the current work. Future research should therefore not only aim to provide more robust and comprehensive data but also address these methodological issues through longitudinal and controlled studies that can evaluate both the improvement of communication skills and long-term outcomes in the training of residents.

## Conclusions

Clinical simulation is a valuable tool in the training of resident nurses in mental health, especially in the management of patients with suicidal behavior. In this study, the effects of this type of training on improving the communication skills of health professionals, allowing them to address high-stress situations with greater efficiency and empathy, were analyzed. The implications of these findings are considerable. An improvement in the communicative capacity of resident nurses not only increases the quality of care provided to patients in crisis but can also contribute to a reduction in suicide rates by providing timely and adequate intervention. However, it is important to recognize the limitations of this study. The available evidence, although promising, remains insufficient to make definitive statements about the effectiveness of clinical simulation in this context.

Despite these limitations, clinical simulation represents a valuable opportunity to enrich the training of resident nurses. To maximize the effectiveness of simulations, it is necessary to develop training programs that are tailored to the specific needs of professionals in training and include continuous evaluations and constructive feedback.

## Data Availability

The raw data supporting the conclusions of this article will be made available by the authors, without undue reservation.
